# Prevalence of low bone formation in untreated patients with osteoporosis

**DOI:** 10.1371/journal.pone.0271555

**Published:** 2022-07-19

**Authors:** Hartmut H. Malluche, Daniel L. Davenport, Florence Lima, Marie-Claude Monier-Faugere

**Affiliations:** 1 Division of Nephrology, Department of Medicine, Bone and Mineral Metabolism, University of Kentucky, Lexington, Kentucky, United States of America; 2 Division of Healthcare Outcomes and Optimal Patient Services, Department of Surgery, University of Kentucky, Lexington, Kentucky, United States of America; University of Vermont, UNITED STATES

## Abstract

**Background:**

Osteoporosis treatment usually starts with an antiresorber and switches to an anabolic agent if it fails. It is known that suppressing bone resorption also results in reduced bone formation. In addition, patients with prior treatment with antiresorbers may have reduced response to subsequent anabolic treatment. This study determined the prevalence of low bone formation in untreated osteoporosis patients to identify patients who may not be optimally treated under the current paradigm.

**Methods:**

This is a cross-sectional study of bone samples stored in the Kentucky Bone Registry. Included samples were from adult patients presenting for workup of osteoporosis. Exclusion criteria were other diseases or treatments affecting bone. Patients underwent iliac crest bone biopsies after tetracycline labeling for identification of bone formation.

**Results:**

107 patients met study criteria, 92 White and 5 Black women and 10 White men. Forty percent of patients (43/107) had low bone formation/bone surface (BFR/BS < 0.56 mm^3^/cm^2^/yr). Clinical and serum parameters did not differ between formation groups, except for type II diabetes, which was found exclusively in the low formation group.

**Conclusions:**

Starting treatment of osteoporotic patients with an antiresorber in all patients appears not optimal for a significant portion.

## Introduction

Osteoporosis is a pervasive health problem of major health and economic impact affecting more than 200 million people worldwide. One in three women and one in five men over age 50 worldwide will experience osteoporotic fractures [1]. Hospital admissions for osteoporotic fractures exceed those of heart attacks, strokes and breast cancer combined [2]. These fractures cause severe pain, long-term disability and early death [3, 4]. Current costs for osteoporotic fractures are estimated at $17 Billion annually in the U.S. [5] and are expected to result in direct costs of more than $25 billion in the year 2025 [6].

Osteoporosis is commonly considered a disease associated with menopause. Estrogen deficiency related bone loss is characterized by increased bone resorption without commensurate increase in bone formation [7–11]. In contrast, age-related bone loss is primarily due to decreased bone formation resulting in deficient replacement of previously resorbed bone [5, 12]. It occurs in both men and women, starting as early as in the fourth decade of life and continues with age [13]. No matter whether it is age, menopause or other reasons for bone loss, this paper addresses the heterogeneity of histologic presentation based primarily on surface-based bone formation rate. Some patients may present mainly with low bone formation which may call for avoidance of the current primary therapeutic approach, i.e., use of an antiresorber.

Antiresorbers are known to suppress bone formation along with resorption. Therefore, it is important to know the bone status of the patient presenting with osteoporosis and avoid treatment that would have a negative effect on bone formation. There are no current data on the bone formation status of osteoporotic patients presenting for treatment. The aim of this study was to determine the distribution of bone formation status in patients presenting for management of osteoporosis using the gold standard for definitive assessment, bone biopsy after tetracycline double labeling.

## Methods

### Patients

This is a retrospective cross-sectional study of bone samples stored in the Kentucky Bone Repository from patients and agreeing to anterior iliac crest bone biopsies between 1995 and 2019. At time of biopsy, all patients signed a written informed consent form approved by the Institutional Review Board of the University of Kentucky (# 95/19-0233). All procedures performed in studies involving human participants were in accordance with the ethical standards of the institutional and/or national research committee and with the 1964 Helsinki declaration and its later amendments or comparable ethical standards. Clinical and biochemical data were obtained from the clinical chart at time of biopsy and stored in the registry. These data were used to select the samples for the current study. Inclusion criteria included; treatment naïve patients presenting for workup of osteoporosis with low bone mineral density by DXA (T-score ≤ -2.5 at the total hip or spine) and/or presence of fragility fractures, adult patients (age above 21 years). The exclusion criteria were genetic diseases (such as osteogenesis imperfecta, hypophosphatemic rickets, etc.), chronic kidney or liver diseases, primary hyperparathyroidism, neoplasms, or previous treatment with medications affecting bone. Histomorphometry was performed on newly cut undecalcified bone sections from the included patients.

### Bone biopsies, mineral bone histology and bone histomorphometry

Prior to biopsy, patients received oral demeclocycline hydrochloride (300 mg) twice daily for 2 days, followed by a 10-day tetracycline-free interval and a course of tetracycline hydrochloride (250 mg) twice daily for 4 days. Anterior iliac crest bone biopsies using the vertical approach were performed under local anesthesia after an additional 4 days. Iliac crest bone samples were processed without removal of the mineral as described previously [14]. Bone histology based on samples from the iliac crest has been shown to be representative for the entire skeleton [15–17].

Two bone samples from each patient of at least 3 mm in diameter and at least 2 cm in length were obtained from the sample stored in the registry and used for histomorphometry. Bone histomorphometry for static and dynamic parameters of bone structure, formation, and resorption was done at a magnification of ×200 using the Osteoplan II system (Kontron, München, Germany). Measurements were done at standardized sites in deep cancellous bone. Bone formation was measured by bone formation rate/ bone surface (BFR/BS). Patients with BFR/BS < 0.56 mm^3^/cm^2^/yr were classified as low bone formation based on published normal values [14], and those with higher values classified as non-low formation. All histomorphometric parameters are in compliance with the recommendations of the nomenclature committee of the American Society of Bone and Mineral Research [18, 19].

### Statistical analysis

Data recorded for the analysis were age, race, gender, body mass index (BMI), menopause status, hormone replacement therapy (HRT), diabetes status, bone mineral density (BMD, T-score), occurrence of fractures, smoking, ethanol consumption, exercise, serum levels of calcium, phosphorus, creatinine, parathyroid hormone, bone specific alkaline phosphatase, osteocalcin, N-terminal telopeptide, and 25 (OH) vitamin D.

Results of categorical variables are presented as number (percentage). Data from continuous variables are given as median (95% confidence intervals) in tables and as median (25^th^ and 75^th^ percentiles) in figures. Distribution between the bone formation groups was assessed using Fisher’s exact or chi-square analyses. Comparisons of continuous variables between groups were performed using independent-sample Mann-Whitney U tests. SPSS^®^ version 25 (IBM^®^ Corp., Armonk, NY) was used for all statistical calculations.

## Results

**Fig 1:**
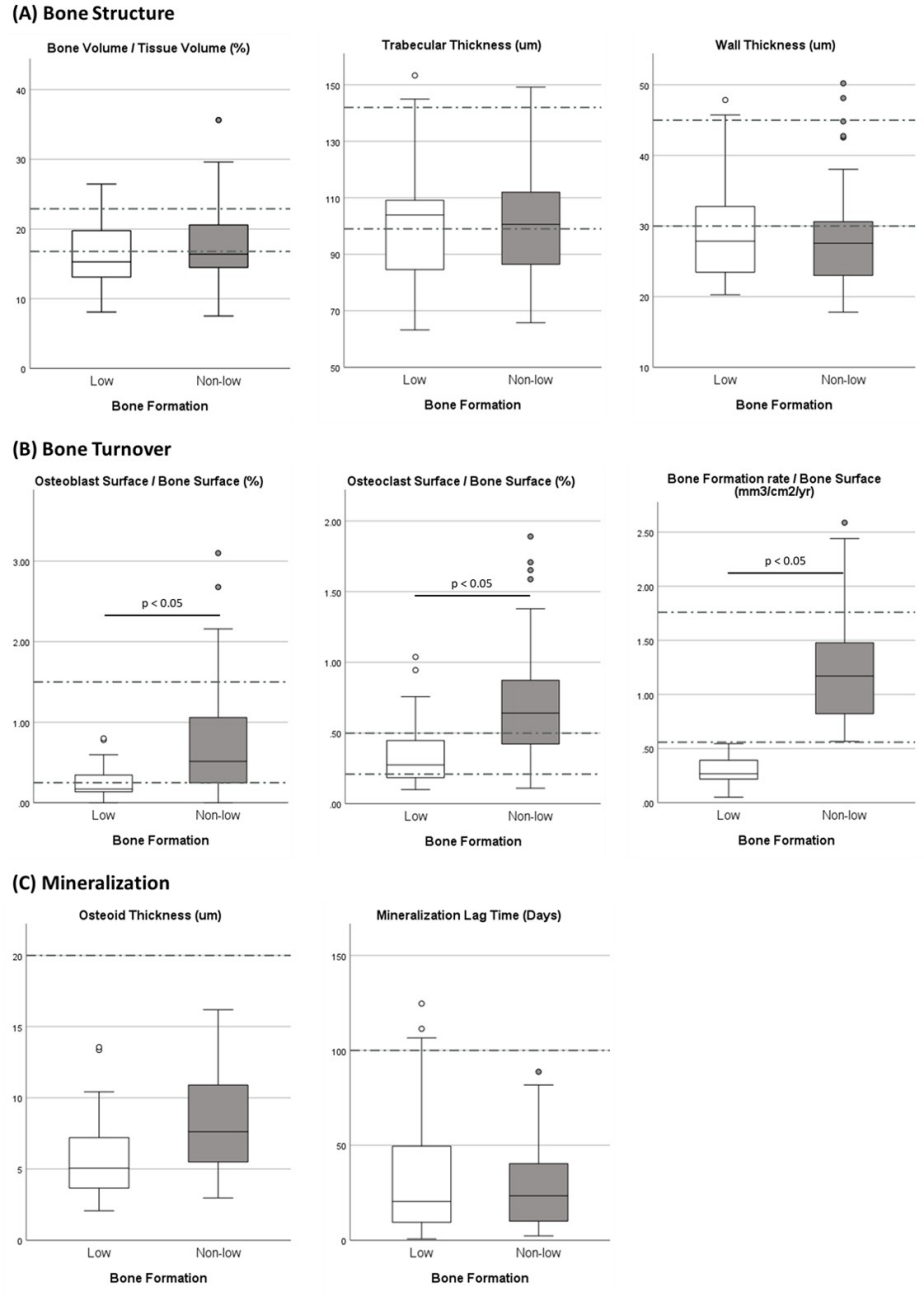
Boxplots of bone histomorphometric parameters of patients with low or non-low bone formation. Dashed grey lines denote normal ranges. **(A)** Parameters of bone structure. **(B)** Parameters of bone turnover. **(C)** Parameters of mineralization.

**Fig 2:**
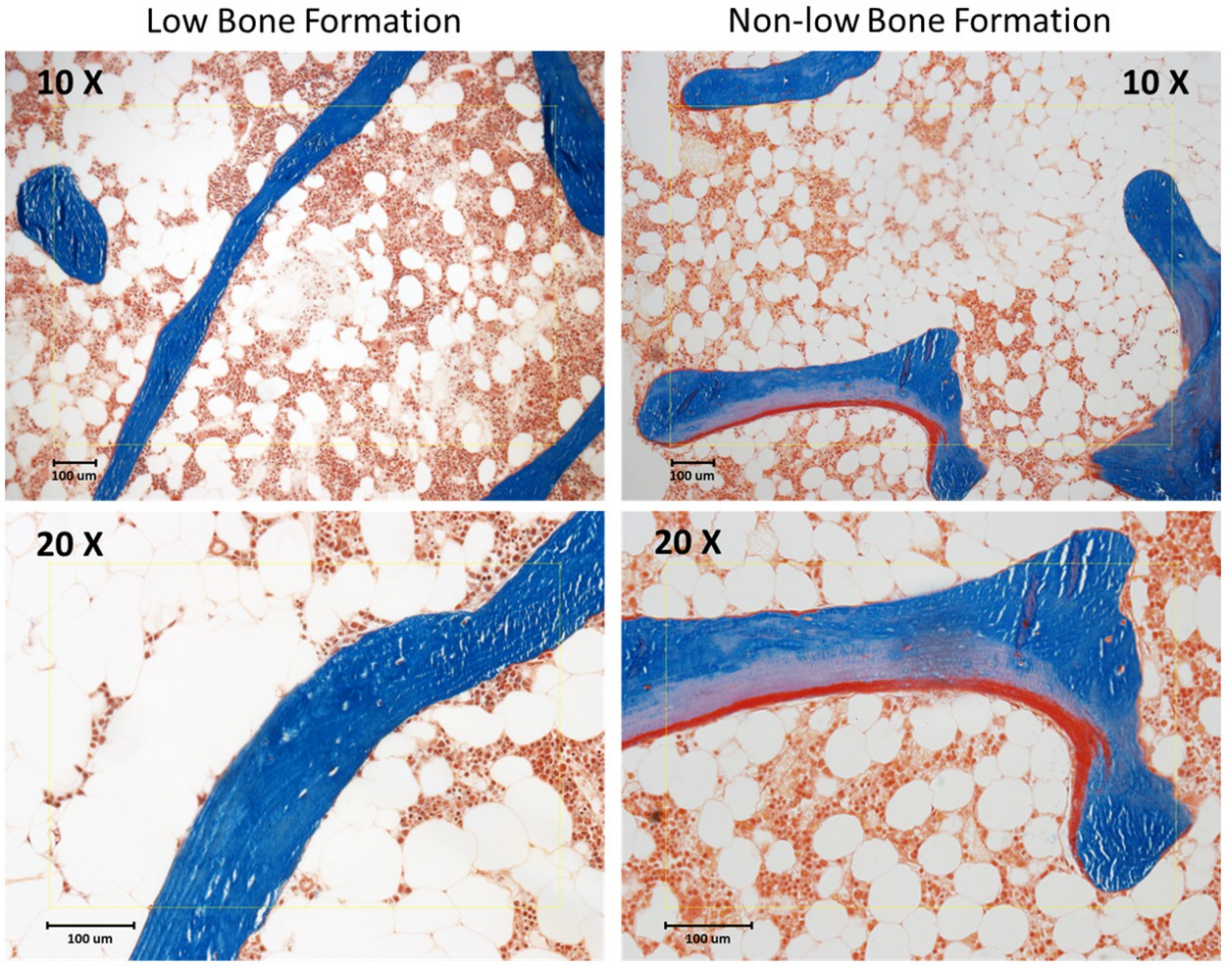
Example of low and non-low bone formation under bright field light microscopy. Microphotographs of undecalcified section of cancellous bone from human anterior iliac crest. Section thickness 4 μm. Masson Goldner Stain. Calcified bone stained blue, osteoid red. **Left panel:** Low bone formation: decreased trabecular bone volume, increased trabecular separation. No surface resorption or osteoid deposition. **Right panel:** Non-low bone formation: decreased trabecular bone volume, increased trabecular separation, thin osteoid seams, increased trabecular surface resorption.

**Fig 3:**
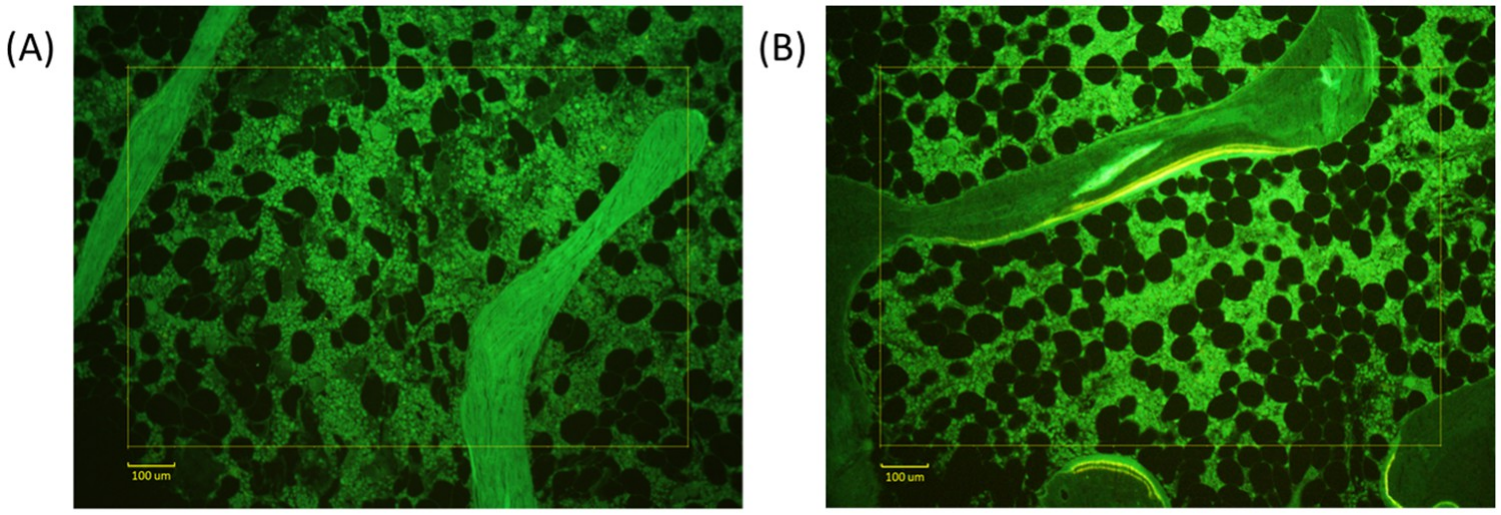
Example of low and non-low bone formation under fluorescent microscopy. Microphotograph of unstained bone section of the same patient viewed under fluorescent light after double tetracycline labeling. Section thickness 7 μm. Magnification 10X. **(A)** In low bone formation, no evidence of tetracycline uptake. **(B)** In non-low bone formation, apposition sites exhibiting double labeling.

A total of 107 patients in the registry met our inclusion criteria, 92 White women, 5 Black women, and 10 White men. Forty-percent (43/107) had low bone formation (Table 1). The small number of Black women were evenly distributed between formation groups. Six of the 10 men (60%) had low bone formation. Type 2 diabetics all had low bone formation. Exercise, smoking, HRT, and alcohol consumption did not vary between formation groups. There was no recognizable difference in fracture history between formation groups. Bone mineral density was lower at the hip in non-low formation patients than low formation (median T-scores -2.25 vs. -1.90, p < .05, Table 1). All serum parameters measured did not differ significantly between groups (Table 2). Histomorphometric osteoclast and osteoblast parameters were higher in the non-low formation group (Fig 1B). There was no evidence of a mineralization defect or osteomalacia in either group (Fig 1C). Microphotographic representations of the human iliac crest biopsy with low and non-low bone formation are shown in Figs 2 and 3.

**Table 1:** Clinical characteristics of patients with different bone formation rates in trabecular bone.

	Low Bone Formation	Non-low Bone Formation
N	43 (40%)	64 (60%)
**Gender**		
Female/Male	37/6	60/4
**Race**		
Whites/Blacks	41/2	61/3
**Age** (yrs)	56 (52, 64)	55 (53, 59)
**Body Mass Index (%)**		
Underweight	2	9
Normal	43	53
Overweight	25	24
Obese	30	14
**Post-Menopause (%)**	84	75
**HRT (% of women)**	41	33
**Type 2 Diabetes (%)**	21 ^**A**^	0 ^**B**^
**Alcohol (%)**		
Yes	23	14
No	70	80
In the past	7	6
**Exercise (%)**		
Regular	23	37
None	77	63
**Smoking (%)**		
Yes	12	19
No	72	59
In the past	16	22
**Fractures (%)**		
Spine	26	25
Hip	9	8
Other	58	45
Total	74	64
**BMD (T-score)**		
Hip	-1.90 (-2.20, -1.00) ^**A**^	-2.25 (-2.50, -1.80) ^**B**^
Lumbar spine	-1.70 (-2.10, -1.10)	-2.30 (-2.40, -1.80)

Results are given as median (95% confidence intervals).

Superscripts ^**A**^ and ^**B**^ indicate group differences significant at p <0.05.

**Table 2:** Serum biochemical parameters of patients with different bone formation rates in trabecular bone.

	Low Bone Formation	Non-low Bone Formation	Reference range
**Serum Calcium (mg/dL)**	9.60 (9.50, 9.80)	9.40 (9.30, 9.70)	8.9–10.2
**Serum Phosphorus (mg/dL)**	3.50 (3.20, 3.80)	3.70 (3.50, 4.00)	2.5–4.5
**Serum Creatinine (mg/dL)**	0.80 (0.78, 0.90)	0.80 (0.74, 0.89)	0.60–1.10
**Serum Parathyroid Hormone (pg/mL)**	38.0 (28.0, 49.0)	34.0 (30.0, 38.0)	12.0–72.0
**Serum Bone-Specific Alkaline Phosphatase (μg/L)**	15.9 (11.8, 17.6)	16.4 (13.0, 19.9)	7.0–22.0
**Serum Osteocalcin (ng/mL)**	13.0 (10.8, 19.8)	15.0 (9.40, 22.0)	8.0–32.0
**Serum N-Terminal Telopeptide (nM BCE)**	13.9 (11.5, 18.7)	15.7 (11.8, 18.0)	6.2–19.0
**Serum Calcidiol (ng/mL)**	38.5 (31.9, 44.0)	34.0 (28.5, 43.0)	30.0–80.0

Results are given as median (95% confidence intervals).

## Discussion

The major finding of our data is that a significant proportion (40%) of patients presenting for workup of osteoporosis have low bone formation and would not be optimally treated using the current antiresorber-driven paradigm. The current situation is that insurance coverage requires starting every osteoporosis patient on an antiresorber, mainly bisphosphonates, then proceed to an anabolic if treatment fails. There is evidence relative to fracture risk that antiresorbers are less effective in patients with low bone formation markers. Bauer *et al*. have analyzed the response of bone to alendronate depending on levels of a bone formation marker [20]. They found that patients in the lower tertile had an inferior reduction in fracture risk with alendronate treatment than those in the higher tertile. Alendronate was no longer significantly better than placebo in reducing fracture risk in the lower tertile patients [20, 21]. This standard of practice is also concerning given that starting with an antiresorber, then switching to an anabolic, makes the anabolic less effective [22–24]. The long skeletal half-life of bisphosphonates, the most frequently used antiresorbers, adds further concern. Our results may provide an explanation for the incomplete overall success of the current osteoporosis treatment paradigm [25].

Our data confirm prior observations of different levels of bone formation in patients with osteoporosis [26–28]. Our rate of low bone formation is higher than found in earlier reports, possibly due to differences in menopausal status, or other population differences including changes in vitamin D and calcium consumption over the decades. However, we could not find a recognizable trend of bone formation during the years of the study. We and others have shown that anterior iliac crest bone histology results are representative for the spine and femur; sites that are predominantly used for clinical diagnosis of osteoporosis [15–17].

Type 2 diabetes was exclusively prevalent in the low formation group. This is in keeping with other reports showing low bone formation in Type 2 diabetes diagnosed by bone markers and bone histology [29–34]. Also, diabetic patients are known to fracture at higher T-scores, providing an explanation for the slightly higher T-scores in the low formation group [35, 36].

The approach to use antiresorptive therapies in patients with high bone turnover markers and anabolic therapies in patients with low turnover markers is not supported by available results or clinical trials [37]. In the fracture intervention trial alendronate treatment was more effective in reducing non vertebral fractures in women with higher P1NP but this was not found for other bone turnover markers [38]. Also, baseline bone turnover markers did not predict facture benefit with teriparatide [39]. However, generally, low P1NP is associated with lower rates of bone loss and less response to zoledronic acid [37]. None of the serum biochemical measures differed between formation groups in keeping with prior studies [40]. An accurate non-invasive diagnostic tool for bone formation is clearly desirable given the challenges of bone biopsy. Ongoing work in our and other laboratories continues to evaluate novel markers for their ability to predict bone formation and resorption. An alternative approach is to train physicians who see patients with osteoporosis in the minimally invasive bone biopsy technique used by hematologists for workup of bone marrow abnormalities [14, 41].

The presented data clearly describe the important differences in bone formation states in osteoporosis patients requesting consultation regarding optimal therapeutic approach. Our hypothesis addresses the need for anabolic agent in patient with low bone formation. It does not postulate that an anabolic agent is ineffective in patient with higher turnover. We posit that the expensive anabolic agent is not needed in the high turnover patient but beneficial for patients with low turnover.

Limitations are related to the cross-sectional nature of the study and no measurements of P1NP and CTX which are recommended by International Osteoporosis Foundation (IOF) and the International Federation of Clinical Chemistry and Laboratory Medicine (IFCC) [42–44]. However, the strength is the rather large sample size of treatment naïve bone biopsies available from our registry.

In conclusion, patients presenting for workup of osteoporosis present with different bone formation states and treatment should be targeted to these differences to be most effective. Starting treatment of osteoporosis with an antiresorber in all patients appears undesirable for a large portion of them. There is great need for development of a non-invasive test for bone formation. As long as such a test is unavailable, the minimally invasive bone biopsy technique used routinely by hematologists can be used for determination of formation.

## Supporting information

S1 Data (XLSX)
